# Elementary Classroom Teachers’ Self-Reported Use of Movement Integration Products and Perceived Facilitators and Barriers Related to Product Use

**DOI:** 10.3390/children7090143

**Published:** 2020-09-18

**Authors:** Roddrick Dugger, Aaron Rafferty, Ethan Hunt, Michael Beets, Collin Webster, Brian Chen, Jeff Rehling, Robert Glenn Weaver

**Affiliations:** 1 Department of Exercise Science, Arnold School of Public Health, University of South Carolina, Columbia, SC 29208, USA; duggerr@email.sc.edu (R.D.); aaron.rafferty8@gmail.com (A.R.); ethunt@email.sc.edu (E.H.); beets@mailbox.sc.edu (M.B.); 2 Department of Physical Education, College of Education, University of South Carolina, Columbia, SC 29208, USA; websterc@mailbox.sc.edu; 3 Department of Health Services and Policy Management, Arnold School of Public Health, University of South Carolina Columbia, SC 29208, USA; bchen@mailbox.sc.edu; 4 Department of Marketing, Moore School of Business, University of South Carolina, Columbia, SC 29208, USA; rehlingj@mailbox.sc.edu

**Keywords:** active lesson, activity break, physical activity, school, children

## Abstract

Movement integration (MI) products are designed to provide children with physical activity during general education classroom time. The purpose of this study was to examine elementary classroom teachers’ self-reported use of MI products and subsequent perceptions of the facilitators of and barriers to MI product use. This study utilized a mixed-methods design. Elementary classroom teachers (n = 40) at four schools each tested four of six common MI products in their classroom for one week. Teachers completed a daily diary, documenting duration and frequency of product use. Following each product test, focus groups were conducted with teachers to assess facilitators and barriers. MI product use lasted for 11.2 (Standard Deviation (SD) = 7.5) min/occasion and MI products were used 4.1 (SD = 3.5) times/week on average. Activity Bursts in the Classroom for Fitness, GoNoodle, and Physical Activity Across the Curriculum were most frequently used. Facilitators of and barriers to MI product use were identified within three central areas—logistics, alignment with teaching goals, and student needs and interests. Teachers were receptive to MI products and used them frequently throughout the week. When considering the adoption of MI products, teachers, administrators, and policy makers should consider products that are readily usable, align with teaching goals, and are consistent with student needs and interests.

## 1. Introduction

Physical activity (PA)—any bodily movement produced by skeletal muscles that requires energy expenditure—has numerous health benefits for children [1]. Consistent PA may help children build strong bones and muscles, improve cardiorespiratory fitness, control weight, reduce symptoms of anxiety and depression, and reduce the risk of developing chronic health conditions [2]. To achieve these benefits, the World Health Organization (WHO) recommends that children (5–17 years) accumulate at least 60 min of moderate-to-vigorous physical activity per day [3]. Despite this recommendation, less than one-quarter of children in the United States participate in 60 min of physical activity daily [2]. Physical inactivity can increase the risk of becoming overweight or obese and increase risk factors for cardiovascular disease, diabetes, and some cancers [2]. The immense cost of physical inactivity necessitates the implementation of effective physical activity promotion among youth.

Schools are an ideal setting for promoting PA because most children spend seven or more hours per day at school [4,5], schools employ personnel trained to work with children, and a variety of school day segments have the potential to provide health enhancing levels of PA (e.g., recess and physical education). In fact, the National Academy of Medicine (formerly the Institute of Medicine) recommends that children accumulate half of their recommended 60 min of moderate-to-vigorous physical activity during school hours [3,6]. Further, schools have been called upon to limit children’s sedentary time during their hours of operation [6,7,8,9]. Despite this promise and these guidelines, schools are not meeting their potential, as physical education and recess are offered less frequently than ever before due to increasing pressure on schools to prioritize academics through high-stakes testing [5,10]. For this reason and as part of a comprehensive school physical activity program [11], elementary classroom teachers are encouraged to implement PA opportunities within general education classroom time [12], a practice referred to as movement integration (MI). MI has been defined as “the incorporation of physical activity, at any level of intensity, within general education classrooms during normal classroom time (p. 691) [13]”.

MI is a multidimensional construct that can be understood in terms of who or what leads PA experiences (e.g., a teacher, student, and technology); what strategies are used to promote movement opportunities (e.g., teaching academic content with integrated PA, integrating activity breaks between academic lessons, and using special equipment such as pedal desks); the nature and extent to which movement is integrated into classroom routines (e.g., when MI is used and how frequently movement opportunities are provided); and the resources used to develop, structure, and guide MI (e.g., pre-packaged materials, curricula, and programs) [13,14]. Concerning MI resources, a wide variety of MI products have been created and tested in the context of interventions designed to increase teachers’ use of MI and children’s PA but studies report relatively low implementation of MI by classroom teachers [15,16,17,18,19]. Several factors including a lack of time, space, and administrative support [20,21,22] may contribute to low implementation of MI in classrooms.

A greater understanding of teachers’ perceptions of the facilitators of and barriers to MI use is needed. While considerable research has addressed teachers’ perceived MI facilitators and barriers [14], previous studies are limited in that they have either examined the use of MI as a general concept (i.e., what would make teachers more likely to infuse PA into their classroom routines) [23,24,25,26,27,28,29], or have been conducted in relation to a single MI product (i.e., what makes teachers more likely to implement a specific MI product, such as Take10) [17]. Therefore, a study that allows teachers to test multiple products in their classrooms is warranted. To do this, the present study adopted a product testing approach. Product testing protocols have been used in consumer-based marketing research to understand the reasons for the high failure rates of new products. Product testing is essential and can help to produce superior products, provide guidance for research and development of new products or improvement of existing products, and identify key components of a product that consumers desire [30,31].

### Purpose and Research Questions

The purpose of this study was to examine elementary classroom teachers’ self-reported use of different MI products and to identify the teachers’ perceived facilitators and barriers related to product use. The following research questions were developed to guide this study:

(1) What is the frequency and duration of teachers’ use of six common MI products?
(2) What barriers to, and facilitators of, MI product usage do teachers perceive?
(3) What is the perceived utility, ease of use, and overall satisfaction with MI products among teachers?
(4) How do teachers perceive student satisfaction with MI products?
(5) How do barriers to, and facilitators of, utility, ease of use, and teacher and student satisfaction with MI products relate to MI product use?

## 2. Materials and Methods

### 2.1. Setting, Recruitment, and Participants

All procedures were approved by the lead author’s university institutional review board prior to the enrollment of the first participant. All subjects gave their informed consent for inclusion before they participated in this study. This study was conducted in accordance with the Declaration of Helsinki, and the protocol was approved by the Institutional Review Board of the University of South Carolina in accordance with 45 CFR 46.101(b)(1) on 7/17/2017 (project: Testing Products to Increase Children’s Physically Active in the Classroom: Identifying What Works and What Does Not). This study took place in four elementary schools in one southeastern U.S. state. All general education classroom teachers at the participating schools were eligible to participate. Teachers were recruited at faculty meetings in the 2018 fall academic semester and participated during the subsequent spring and fall academic semesters of 2019. A total of 30 of 58 teachers who consented were randomly selected to participate in this study. In the fall of 2019, teachers who withdrew from this study or changed schools (n = 12) were replaced with teachers at the same school who taught the same grade (n = 10), with a total of 40 teachers participating in this study. Reasons teachers left this study included withdrawing from this study (n = 4), transitioning to a new school (n = 6), retirement (n = 1), and maternity leave (n = 1).

### 2.2. Study Design/Procedure

This study employed an explanatory mixed-methods design [32] and product testing protocols [30,31] to quantitatively assess MI product usage and qualitatively assess teachers’ perceptions of barriers to, and facilitators of, MI product usage. Each teacher tested a total of 4 MI products in their classrooms over 4 separate 5 day product tests (i.e., 5 days for each product) that were separated by at least two weeks. To prevent contamination, all teachers at a given school tested the same product during the 5 day product testing period. Each MI product contained its own orientation and respective training materials which were provided to teachers the week prior to the 5 day product test. The teachers were instructed to use the products however, whenever, and as often as they saw fit. All teachers were informed of this study’s purpose and that the research staff were not affiliated with the MI products.

### 2.3. Product Descriptions

A list of the products and product features is provided in Table 1. Products were selected in order to (1) include products that were widely available to teachers, and (2) represent a broad range of attributes offered by current MI products.

Move for Thought (MFT). Move for thought is a free online resource developed by researchers that provides a virtual community of practice designed to help classroom teachers integrate movement into classroom time. Teachers are required to create a profile on the website in order to access videos, links, and a blog that allows users to share MI experiences and ideas [33]. This resource contains grade-specific activities that include both academic-integrated and non-academic-integrated MI options.

Activity Bursts in the Classroom for Fitness (ABC). ABC, developed by researchers and teachers, was designed to reduce the time teachers spend in behavior management by converting that time into organized bursts of physical activity [34]. It provides academic integrated movement activities that teachers can incorporate into English-language arts, math, and social studies lessons. ABC is not grade-specific; rather, its guidebook/manual includes physical activity bursts lasting no more than 5 min each. Activity bursts have three components—A: warm up, B: core activity (e.g., exercise and stretching), and C: cool down.

Take10. Take10 is a classroom-based guidebook developed by researchers that provides teachers with grade-specific lesson plans that are integrated with physical activity [35]. Take10 includes activity-infused lesson plans for children in K–5th grade in the areas of English-language arts, health, math, science, and social studies. Activities incorporate a warm-up, activity, cool-down, and nutrition questions designed to last for a maximum of ten minutes [35]. Take10 costs $85 (grades 1st, 4th, 5th) or $112 (K, 2nd, 3rd) per guidebook/manual.

GoNoodlePlus. GoNoodle is a free online resource developed by a team of professionals in education, youth entertainment, and product development. It contains a series of videos either providing activity breaks or integrating grade-specific English-language arts, health, math, science, and social studies academic content that integrate various physical activities (e.g., dance, exercise, and yoga). Videos last for approximately 5–10 min, are set to music, led by avatars, and incorporate elements of social and emotional wellness to support child development [36]. For $99 per year, GoNoodlePlus offers additional videos, printable learning extensions that are directly related to video content, and online and printable assessments. Teachers were provided with GoNoodlePlus during product testing.

Instant Recess. Instant Recess is a DVD series developed by researchers that includes physical activity breaks intended for a variety of settings including workplaces, schools, and churches. Instant Recess helps create opportunities for participants to engage in 10 min bouts of activity [37]. In the videos, a dance instructor leads viewers through a variety of dances and exercise movements from around the world, which develops cultural awareness. There are no grade level specific activities and the 11 DVD set costs $99.

Physical Activity Across the Curriculum (PAAC). PAAC, also developed by researchers and teachers, is a free guidebook/manual that provides a series of lesson plans which integrate physical activity into English-language arts, health, math, science, and social studies academic content. Lesson plans were developed by merging existing teacher lessons with Take10 curriculum activities [38]. Lessons are presented in a 3-ring notebook with specific objectives and required materials. The developers suggest engaging children in 10 min bouts of activity twice per day [17].

### 2.4. Quantitative Measure

Daily Diary. For each day of product testing, teachers completed a brief survey identifying the amount of time spent planning for MI product use and time and duration of use (Appendix B). For this study, use was defined as classroom-instructional time spent delivering content from the MI product to students to increase physical activity. Planning time was defined as non-classroom instructional time teachers spent preparing to incorporate MI product content into their lesson plans. If the MI product was not used on a day, teachers identified the reason they did not use the product. Appendix A contains the daily diary.

### 2.5. Qualitative Methods

Focus Groups. After each round of product testing, teachers participated in a focus group held at their respective schools. All focus groups occurred the week following the product testing in a private room after school and lasted no longer than 20 min. Focus groups were facilitated by trained research assistants using a semi-structured interview guide (Appendix C) and were recorded with the permission of teachers. Interview questions were informed by product testing methods and techniques [30,31], previous MI survey research [27,28,39,40,41], published reviews of MI and school-based PA promotion [13,42,43], studies of MI resources/strategies [27,28,38,44,45,46,47,48,49] and theoretical implementation frameworks [50,51,52]. Interviewers used iterative, probing questioning tactics to encourage participants to share differing points of view and to elicit honesty in responses. Questions centered around: teacher perceptions of student feelings about the product, ease of use, familiarity with the product, reasons for non-use, and overall satisfaction with the product. Follow-up, individual interviews were held with teachers who were not present at focus group discussions.

### 2.6. Data Analysis

Quantitative Analyses. Descriptive statistics (i.e., frequency, means, and standard deviations, where appropriate) were calculated for all survey responses using Stata (v16, StataCorp LLC, College Station, TX, USA).

Qualitative Analysis and Trustworthiness. All focus group discussions and teacher interviews were transcribed and imported into NVIVO 12 software. Data analysis was inductive in nature and was guided by grounded theory [53] and an immersion crystallization approach [54] using a three-step latent coding technique [55]. First, coders independently read and generated codes for a single transcript by grouping recurring words, phrases, and themes. Second, coders and a third reviewer met in order to review codes, integrate/add codes to a running list of codes generated from each transcript (i.e., coding guide), and to arbitrate any disagreements between coders. Third, transcripts were revisited by the coders to determine if additional codes were needed and if the coding guide had reached saturation [53]. This process was repeated until all transcripts were read and a comprehensive list of codes was generated. Once a final list of codes was agreed upon it was used as a coding guide to review and code all focus group transcripts.

Codes were classified into three broad level themes (e.g., logistics, alignment with teaching goals, and student needs and interests). The two independent coders and third reviewer combined and classified codes into subthemes through discussion. Themes were developed using inductive analysis and were defined as either a facilitator of or barrier to MI product use. Themes were considered a facilitator of use if teacher responses indicated that it enabled use while themes were considered a barrier if teachers indicated that it hindered use. Given this study’s focus on identifying characteristics of MI products that could lead to teachers’ product use and adoption, themes were subsequently analyzed for extensiveness and frequency [56]. Extensiveness refers to the number of schools represented for a particular theme while frequency refers to the number of teachers represented for a particular theme. Themes that were identified at ≥50% of schools and ≥25% of teachers are included in the findings [56].

Data Confidentiality. Focus group transcripts were recorded and stored on a password-protected computer and kept in a locked office. Only three of the co-authors (R.G.W., A.R., and R.D.) had access to the recordings. Additionally, teacher data was de-identified by assigning a numeric code identifier to the transcripts.

### 2.7. Trustworthiness of Findings

Several steps were taken to ensure the trustworthiness of the study findings. First, as a form of peer debriefing, themes were reviewed by and discussed with two colleagues in the field of school-based physical activity research who were not involved in theme generation or coding [57]. Second, we employed synthesized member checking protocols [58]. This involved asking the teachers to review and confirm the qualitative findings. A total of five teachers participated in member checking. The low response may be a result of school closures due to the COVID-19 pandemic. Finally, data were triangulated from multiple sources including daily diaries of product use and focus groups [59].

## 3. Results

### 3.1. Quantitative Findings

Table 2 presents school- and teacher-level demographics. Table 3 displays product use data gathered from all daily diaries. Overall, teachers used MI products an average of 4.1 (Standard Deviation (SD) = 3.5) times per week. ABC for fitness was the most commonly used MI product with an average of 5.7 (SD = 6.1) uses per week while MFT was the least commonly used product with an average of 1.4 (SD = 1.9) uses per week. Overall planning for each MI product use lasted 6.6 (SD = 8.5) min. Planning time ranged from 3.2 min (SD = 3.3) for GoNoodlePlus to 14.1 min (SD = 7.6) for MFT. Overall, the average duration of each MI product use was 11.2 (SD = 7.5) min. Duration of use ranged from 6.3 (SD = 0.04) minutes for GoNoodlePlus to 13.3 (SD = 3.8) minutes for MFT. The most commonly cited reasons for not using an MI product were a lack of time (41%), technical difficulties (12%), student dislike of the product (6%), student misbehavior (6%), and lack of space (4%).

### 3.2. Qualitative Findings

#### 3.2.1. Themes

Teachers identified three broad themes as facilitators of or barriers to use of MI products: logistics, alignment with teaching goals, and student needs and interests. The extensiveness and frequency of these themes and their subthemes are presented in Table 4 and discussed in detail below.

#### 3.2.2. Logistics

Teachers identified logistics of using a product as a facilitator of or barrier to MI product use. For this study, logistics refers to contextual factors relating to the planning and delivery of the product (e.g., time to plan or implement). For logistics, four subthemes emerged: time, ease of use, missing features, and alternative for outdoor activities.

### 3.3. Logistics—Time

Teachers commonly referenced a lack of time to prepare as a primary reason for non-use. The amount of time required to read through instructions, select activities, and plan to integrate these activities within a lesson was noted as a barrier to product use. For example, a teacher using ABC’s “Alphabet Fitness Chant” found that this activity required excessive planning time due to a lack of visuals.

*“I would have needed a visual to remember the activity and I didn’t want to keep reading the book. I would have had to write out all of the actions which would have taken an extra ten minutes. So that’s something I would have had to [spend time] preparing prior to”. (Barrier) Teacher #21, Kindergarten, at School D using ABC*

Similarly, a teacher using Take10 noted a lack of time to prepare as a barrier to use.

*“It was just a lot of reading. I have a lot of reading already for 3rd grade standards. So planning it...was a lot of reading”. (Barrier) Teacher #35, 3rd Grade, at School B using Take10*

Some teachers discussed how the duration of product activities was a barrier to use. A teacher using Instant Recess found that the length of the videos caused her students to lose focus.

*“I thought some of it was long. My kids started getting a little [distracted] and a little giggly towards the end because it was longer than they were accustomed to”. (Barrier) Teacher #14, 1st Grade, at School C using Instant Recess*

Alternatively, some products were perceived to require minimal planning time.

*“That’s what I liked, it didn’t require a lot of planning time, you could quickly just go to whatever subject area you were covering and quickly find an activity to engage the students in”. (Facilitator) Teacher #13, 2nd Grade, at School C using PAAC*

A total of 27 teachers at 4 schools referenced time as a facilitator of or barrier to product use. Overall, time was coded as red (i.e., less than one-third of teachers identified time as a facilitator of product use). Specifically, PAAC was coded as green (i.e., greater than two-thirds of teachers identified time as a facilitator of product use), while Take10, ABC, MFT, and Instant Recess were coded as red. Time was never identified as facilitator of or barrier to use of GoNoodlePlus.

### 3.4. Logistics—Ease of Use

MI products that contained clear instructions and visuals were considered easy to use. This was identified as a facilitator of use.

*“The directions were very clear and easy to follow. Easy to read as far as getting the kids to do the activity”. (Facilitator) Teacher #6, 3rd Grade, at School A using Instant Recess*

*“The book had examples if I didn’t know how to do it [it would show you how]. It has pictures and provides the models too”. (Facilitator) Teacher #5, 3rd Grade, at School A using ABC*

Alternatively, products with too much content or unclear activities were not considered easy and this was considered a barrier to use. A teacher describing the ease of use of ABC noted,

*“It had a lot of words which was discouraging. I saw all those words with one picture and that was too much. It would have taken too much time to read and figure out how to incorporate into my school day”. (Barrier) Teacher #23, 4th Grade, at School D using ABC*

Additionally, some teachers found that activities within MFT did not provide sufficient instructions for activities.

*“I did not like the fact that I found myself going to the web to get additional information about the different activities. For example…the activity Bingo...it did not give me enough information on the blog page. I went online to research exactly what it was about; I just needed more detailed information”. (Barrier) Teacher #1, 4th Grade, at School A using MFT*

Some teachers preferred the guidebook/manual format of MI products (ABC, Take10, PAAC) because it was not technology dependent, while other teachers found computer-based products easy to use.

*“It was sitting right there on our desk. We didn’t have to type anything in a link or open a tab on a computer. We could just already have it tabbed on our book and open it”. (Facilitator) Teacher #17, 4th Grade, at School C using PAAC*

*“I used the online access and that format was easy to use. Just click on the link”. (Facilitator) Teacher #6, 3rd Grade, at School A using Instant Recess*

Teachers found some activities to be so simple and easy to remember that they could be implemented without referring to the MI curriculum. One teacher described how PAAC’s “Jump and Spell” activity was so straightforward that she did not need to reference the instructions.

*“They [students] said ‘Can we do Jump and Spell?’ and I said ‘Sure!’ I didn’t have to pull out the [book]; we knew exactly what to do. It was a no brainer”. (Facilitator) Teacher #14, 1st Grade, School C, using PAAC*

### 3.5. Logistics—Missing Features

Teachers indicated that all MI products were lacking at least one essential element that would facilitate use. Some teachers requested that products contain visual aids. For example, a teacher suggested that ABC could benefit from additional visuals.

*“Maybe if you had big pictures, so that you could hold it up and show [students]. That would be helpful”. (Barrier) Teacher #33, Pre-K, at School B using ABC*

Teachers also requested activities that did not require them to lead the class through the activity.

*“One thing I would like to add is, I would like the idea of some of the activities being set up to be student-led”. (Barrier) Teacher #9, 1st Grade, at School A using Take10*

Teachers also lamented the lack of music in products that were guidebooks/manuals (e.g., Take10, ABC, and PAAC).

*“…it would be better if it had music that went along with it”. (Barrier) Teacher #34, 1st Grade, at School B using Take10*

### 3.6. Logistics—Alternative for Outdoor Activities

Some teachers appreciated MI products because they could serve as an alternative option for recess and outdoor activities due to inclement weather or an atypical schedule.

*“We did have a few days where it was cold and we couldn’t [go outside] and I thought it would be a good alternative, and I [used] it more than once”. (Facilitator) Teacher #27, 4th Grade, at School D using Instant Recess*

*“I think it came in handy when we as early childhood teachers could not go outside to have recess due to testing...” (Facilitator) Teacher #2, 1st Grade, at School A using ABC*

### 3.7. Alignment with Teaching Goals

The second theme in the findings was the degree of alignment of an MI product’s content with teaching goals, which captured the extent to which the product meets teacher classroom expectations and learning goals. As with the first theme (logistics), the degree of alignment between MI products and teaching goals acted as either a facilitator of (when alignment was high) or a barrier to (when alignment was low) product use. Alignment with teaching goals consisted of five subthemes: reinforces academics; classroom management; developmental appropriateness; refocus, breaks and transition; and customizable.

#### 3.7.1. Alignment with Teaching Goals—Reinforces Academics

Teachers identified the inclusion of academic content in MI products as facilitator of product use. Some teachers noted they specifically disliked products that did not have academic content (e.g., ABC and Instant Recess) or were not aligned with academic standards.

*“I still give them time to move around but they [MI activities] are usually academic-based. So [students] are singing about the water cycle or something we are learning or doing movement with it. I like brain breaks that are more like that. So that is one thing I did not like about this product”. (Barrier) Teacher #34, 1st Grade, at School B using ABC*

Teachers also valued products and activities that could be adapted to fit the content that they were teaching.

*“I liked that you could integrate it across any type of curriculum that you were teaching”. (Facilitator) Teacher #20, 3rd Grade, at School C using PAAC*

*“I was able to integrate it into my health lessons. We talked about fitness and movement and the importance of exercising. It fit right into it as a lesson, so I was able to incorporate it with health and science”. (Facilitator) Teacher #2, 1st Grade, at School A using ABC*

Teachers noted that MI increased student engagement in the lesson. A teacher described how a spelling activity in the PAAC curriculum combined movement with spelling to promote learning.

*“I think it [PAAC] helped make spelling fun because a lot of kids hate spelling…it made it better for them”. (Facilitator) Teacher #10, 2nd Grade, at School C using PAAC*

Products that combined learning and movement were generally the most acceptable to teachers.

#### 3.7.2. Alignment with Teaching Goals—Classroom Management

Teachers noted that some products assisted with classroom management while others contributed to behavioral disruption. Some products (e.g., GoNoodlePlus and Instant Recess) incorporated music (e.g., GoNoodlePlus and Instant Recess), dance (e.g., GoNoodlePlus and Instant Recess), and activities that required children to jump and move around the classroom. Teachers of younger grades (K–3) found it difficult to settle the children after activity and return to academic instruction.

*“It was really hard to get the kids focused afterwards. It was really hard to get them back focused on what I was actually teaching”. (Barrier) Teacher #34, 1st Grade, at School B using Take10*

Teachers found products more facilitative when the products incorporated cool-down periods (e.g., GoNoodlePlus, MFT, and ABC) or contained calming activities (e.g., yoga, deep breathing— GoNoodlePlus, MFT, and ABC).

*“I found that during testing a lot of emotions came out. Frustrations and things like that. I found that [cool down breathing] was helpful during testing”. (Facilitator) Teacher#35, 3rd Grade, at School B using ABC*

#### 3.7.3. Alignment with Teaching Goals—Developmental Appropriateness

Some activities within the MI products were deemed developmentally inappropriate for certain grade levels. A number of activities were too complex or involved an academic skill that had not been covered. For example, a teacher using the sentence break activity in ABC to teach parts of speech found it to be misaligned with her grade level.

*“I don’t think some of the activities would have benefited them [sentence structure activity] because they don’t know verbs and nouns. They are just learning letters and letter sounds, so that just doesn’t fit their grade”. (Barrier) Teacher #21, Kindergarten, at School D using ABC*

Moreover, some of the movements were too difficult for students (e.g., jumping jacks for Kindergartners) or misaligned with the grade level (e.g., hokey pokey for third grade).

*“Some kids didn’t know how to do jumping jacks or know certain other things”. (Barrier) Teacher #10, 3rd Grade, at School C using Take10*

*“You know the Hokey Pokey’s in there too, but I have third graders so they are too cool for that. They weren’t about to do the Hokey Pokey”. (Barrier) Teacher #32, 1st Grade, at School B using Take10*

#### 3.7.4. Alignment with Teaching Goals—Refocus, Breaks, and Transition

Overall, teachers indicated that products that provided a break for students and/or teachers were facilitative of use. One teacher noted that GoNoodlePlus provided a mental break for her students.

*“I wanted it to be something that was more so a break for them from academics to give them like a down time or just a mental break”. (Facilitator) Teacher #21, Pre-K, at School D using GoNoodlePlus*

Teachers recognized that children need breaks from learning to regain focus.

*“I liked it because I know kids need movement and you can tell when you need a break in teaching when the kids are getting tired”. (Facilitator) Teacher #1, 4th Grade, at School A using ABC*

All teachers utilized the products to provide this break, and some used the MI products as a transition period to prepare for the next activity.

*“It was really easy to use for transitions.” (Facilitator) Teacher #4, Kindergarten, at School A using ABC*

#### 3.7.5. Alignment with Teaching Goals—Customizable

Some MI products contained activities that could be incorporated into a variety of academic subjects (e.g., ABC, GoNoodlePlus, and PAAC). Activities could be adjusted to match skill level and some physical exercises could be modified to accommodate student fitness levels. This level of adaptability was a facilitator of use for teachers because it reduced planning time and supported academic improvement.

*“I like that it [GoNoodlePlus] was customizable for the words that we were studying that week. This was the highest spelling grades we’ve had all year!”. (Facilitator) Teacher #12, 4th Grade, at School C using GoNoodlePlus*

A teacher found that PAAC required minimal planning time and could be applied within multiple academic subjects.

*“I did all of my planning on Friday, six or seven minutes for the whole week. I did this a couple of times with spelling and then I made an adjustment to make [it] work for what we were doing in social studies”. (Facilitator) Teacher #15, 3rd Grade, at School C using PAAC*

#### 3.7.6. Student Needs and Interests

The third theme focused on the extent to which MI products fulfilled student needs and interests. For this study, student needs and interests were defined as the degree of alignment between the MI product and student preferences, classroom culture, and learning needs. Similar to the first two themes, product alignment with the students’ needs and interests was a facilitator of product use while misalignment was a barrier to product use. Student needs and interests included five subthemes: student health benefits; student enjoyment; newness/difference; comfortable, class-specific interests; and movement break.

#### 3.7.7. Student Needs and Interests—Student Health Benefits

Teachers considered MI products to be highly beneficial for their students’ health, noting that their students benefit from the exercise, flexibility training, and overall health knowledge gained through the MI products. Moreover, teachers recognized that children need physical activity to be healthy.

*“I think MFT will be good for our kids because our kids are very active and giving them more chances to move will be better for them in the end”. (Facilitator) Teacher #4, Kindergarten, at School A using MFT*

*“I liked how [ABC] explained to the students what is necessary when you are exercising. That there needs to be a warmup and you need to stretch. You need to be ready to exercise”. (Facilitator) Teacher #36, Kindergarten, at School A using ABC*

A teacher found that an activity in Take10 exposed student’s inadequate fitness levels and provided an opportunity for improvement.

*“…they would have to jump and hop and by the time they were finished they were breathing heavy, but they enjoyed it. I said ‘Sounds like y’all are out of shape; we need to keep doing this”. (Facilitator) Teacher #18, 2nd Grade, at School C using Take10*

#### 3.7.8. Student Needs and Interests—Student Enjoyment

Most teachers felt that their students enjoyed the MI products. Students would frequently request time to participate in MI.

*“It gets them excited for sure. Those three guys. The Fresh Blazers! Anything that they do, they love it! If they could just teach everything”! (Facilitator) Teacher #10, 3rd Grade, at School C using GoNoodlePlus*

*“They would always want to remind me ‘Mrs. [Teacher name] can we do the ABC chant’?”. (Facilitator) Teacher #2, 1st Grade, at School A using ABC*

Teachers perceived that students enjoyed the movement activities so much that students did not realize that they were learning academic content simultaneously.

*“The students are literally thinking we are just having fun and exercising even though they are answering questions…They aren’t even focusing on the academics. They’re thinking ‘Oh man I am having fun’!” (Facilitator) Teacher #1, 4th Grade, at School A using ABC*

Overall, most teachers perceived that students valued the opportunity to be active, regardless of the MI product that was used. However, some products were clearly not enjoyed by all students. Specifically, teachers who tested Instant Recess noted that student dissatisfaction was linked to outdated and less engaging visuals.

*“I would say it did not sustain their attention. As far as the appearance, the visuals, it’s not as right [appealing]”. (Barrier) Teacher #21, Kindergarten, at School D using Instant Recess*

*“I don’t recommend it, it’s just so dated, and the kids are used to technology, and they are going to quickly compare that the visuals don’t match”. (Barrier) Teacher #21, Pre-K, at School D using Instant Recess*

#### 3.7.9. Student Needs and Interests—Newness/Difference

Teachers also valued MI products because they presented academic content in a new and engaging way that was different from what their students were accustomed to.

*“I think they are excited because [MFT] is a new program that we have never seen before. And more activities that we get to do instead of, you know, the regular things I have been doing…they are going to be really engaged because it’s different”. (Facilitator) Teacher #4, Kindergarten, at School A using MFT*

*“It helped make spelling fun because a lot of kids hate spelling. For the kids to be able to punch out the words and make a movement made it better for them. It was just exciting”. (Facilitator) Teacher #10, 3rd Grade, at School C using PAAC*

Additionally, some teachers valued the MI content because it exposed their students to different cultures, dances, and movement patterns. A teacher referencing the yoga activity in MFT said,

*“It was different. My kids have never experienced yoga before, so that was their first time and they were excited about it”. (Facilitator) Teacher #4, Kindergarten, at School A using MFT*

Similarly, the cultural dances and movements showcased in Instant Recess were perceived favorably by both students and teachers.

*“I liked the different genres of music, so it wasn’t just kind of the same thing and it’s something that the kids are not readily exposed to”. (Facilitator) Teacher #6, 3rd Grade, at School A using Instant Recess*

#### 3.7.10. Student Needs and Interests—Familiarity

Products that contained content that aligned with classroom-specific (e.g., teacher or student) activities or routines were valued by teachers. Students were familiar with certain activities/movements because teachers implemented similar activities prior to product testing.

*“I do a lot of activities like this anyway in my class, so [my students] were used to it”. (Facilitator) Teacher #14, 1st Grade, at School C using PAAC*

*“My students enjoyed the fitness breaks. They were movements that they [students] were accustomed to doing, so it wasn’t a chore to do”. (Facilitator) Teacher #24, 3rd Grade, at North Vista using ABC*

Additionally, teachers noted that some products contained games or activities that elicited participation from even the shyest students.

*“I’ve seen some of the kids that are typically kind of shy, they get right into it [movement activity]. They move. They don’t have to speak you know… so they can participate. They feel really comfortable doing it and they love it”. (Facilitator) Teacher #16, 4th Grade, at School C using GoNoodlePlus*

Students from diverse cultural backgrounds valued products that incorporated aspects of their unique culture into the activities (e.g., Instant Recess). Instant Recess videos incorporated cultural dances and movement patterns into their videos. For example, one dance video showcased cultural dances from the Philippines.

*“My kids liked the movement; they liked the dancing and everyone got into something”. (Facilitator) Teacher # 24, 2nd Grade, at School D using Instant Recess*

*“It taught different dance moves and different words that they might use in the Philippines. It was a language my student was familiar with; he brightened up when he saw that”. (Facilitator) Teacher #11, 1st Grade, at School C using Instant Recess*

#### 3.7.11. Student Needs and Interests—Movement Break

Teachers recognized that students enjoyed the products simply because of the movement they provided.

*“My students were very engaged. They enjoyed the kinesthetic movements as we chanted along using the alphabet”. (Facilitator) Teacher #2, 1st Grade, at School A using ABC*

Students were described as avid fans of activities incorporating dance or music.

*“Every time I would say dance break, we all would [sing] ‘da na na na’ and the other kids would say ‘dance break’. They just made up their own little thing and would line up for lunch. That’s how much they really like the little activities”. (Facilitator) Teacher #4, Kindergarten, at School A using ABC*

Moreover, teachers acknowledged students’ need for movement.

*“I did it [used Take10] when I could tell they were starting to get restless, like ‘alright, let’s stand up’. You could tell they were thankful for it. They were tired of just sitting all day but they would rather get up and dance you know”. (Facilitator) Teacher#10, 3rd grade, at Lucy Davis using Take10*

## 4. Discussion

This study examined teachers’ use of MI products and perceptions of facilitators and barriers related to MI product use. The findings from this study can be used to support the adoption of MI by teachers. Overall, teachers considered the MI products they tested as beneficial for students and an asset to their teaching goals. Teachers indicated that MI product logistics were a key facilitator of or barrier to product use. Products that teachers rated highly in terms of logistics were used more frequently. This finding suggests that a key determinant in adoption of MI by teachers is product logistics, which varied by product. Similar to the present study, past research has shown that teachers favor MI products that are quick and simple to use [20].

Teachers likely consider logistic factors to be important because they have limited time. In this study, time to plan and implement MI was among the most frequent and extensive themes identified during focus groups. The increased emphasis on testing and standards-based instruction in schools has left teachers with little time to incorporate additional initiatives such as MI [11,20,24,60]. Teachers preferred MI products that were short in duration and required minimal planning time. The most frequently used products (e.g., PAAC, GoNoodlePlus, and ABC) required minimal planning time and were brief in duration. Conversely, products that were used infrequently required the most planning time (MFT, Take10) and lasted longer (Instant Recess). Moreover, the most commonly cited reason for non-use in teachers’ daily diaries was a lack of time. Other logistic factors (e.g., ease of use and missing features) also correspond to teachers’ need to effectively manage time when using MI products. Prior studies have also found that ease of implementation [20,21,27,61,62], particularly MI strategies that can be completed in a short period of time [22], is important to teachers.

While logistics was the primary factor affecting MI use, other factors also facilitated or hindered MI product use. Teachers preferred and used products that reinforced academic content. This finding aligns with previous studies in which teachers recognized that MI was useful for supplementing and/or reinforcing academic content [20,21,22,27,62]. In the current study, while teachers valued MI products that aligned with their teaching goals (e.g., behavior management, adaptability, and developmental appropriateness), these elements were supportive, but did not appear to discriminate between products that were used more and less frequently. Consistent with the current findings, teachers in previous studies have also cited behavior management [26,60,63] and developmental appropriateness concerns [60] as barriers to use of MI. It is not surprising that teachers perceived that MI products may lead to disruptions and behavior management challenges as research has shown that student misbehavior occurs primarily when students are out of their seats in the classroom [64,65]. Further, teachers who have limited experience and training in managing active environments may perceive times that children are out of their seats in the classroom as out of control. Thus, training on behavior management strategies for teachers may be a critical component of MI products [39,66]. MI products that include cool down activities may also help teachers transition back to more traditional classroom activities without behavior disruptions.

Teachers in the current study overwhelmingly rated the MI products they tested positively on student needs and interests. Consistent with past research, teachers recognized that students enjoy and need the opportunity to move [22,67,68] and some teachers even found it beneficial for student learning [26]. Because these benefits were pervasive across MI products in the present study, but product use varied, it appears that the extent to which a teacher perceives a MI product to meet student needs and interests is a necessary first step for MI product use but is not sufficient to drive teacher use of products.

This study has several strengths. It is one of the first studies to link teacher perceptions of MI barriers and facilitators with actual use of a variety of MI products. This is also one of the first studies to explore MI using a mixed-methods study design. Confidence in the findings are strengthened because teachers tested a variety of MI products (n = 4 per school) with overall high frequency of use (i.e., avg 4.1 uses/week). However, this study is not without limitations. This study used a convenience sample of schools in one southeastern state, and as a result, findings may have limited generalizability. Yet, the findings from the current study align well with previous research indicating that the schools and teachers sampled here may not systematically differ from other schools and teachers. Another possible limitation is that, although the products included in the current study are widely available to teachers and represent a wide range of product characteristics, not all existing MI products were tested. Teacher attrition was also an issue, with 12 teachers withdrawing from this study. There is a chance that the teachers that left this study were systematically different (e.g., teaching experience and age) from the teachers that completed this study and/or the teachers that took their place. Finally, MI use statistics should be interpreted with caution because teacher use of MI over a 5 day product testing period may not accurately reflect use during a full academic year. As teachers gain experience with MI over the course of the year, the amount of time spent planning for use as well as the frequency and duration of use may change.

## 5. Conclusions

In conclusion, for teachers in the present study, MI product logistics were the primary factor affecting MI use. MI products that were short in duration, easy to understand and implement were preferred by teachers. Other factors (e.g., alignment with teaching goals, student needs and interests) facilitated, but did not determine use. We suggest that policy makers, school administrators, and teachers considering adoption of MI select products that are favorable in terms of logistics in order to maximize teacher use and student benefit.

## Figures and Tables

**Table 1:** Movement Integration Product Characteristics.

Characteristic	GoNoodle	PAAC	ABC for Fitness	Take10	Move for Thought	Instant Recess
Format	Web based	Guidebook/manual	Guidebook/manual	Guidebook/manual	Web based	DVDs
Delivery	Technology led	Teacher led	Teacher led	Teacher led	Teacher led	Technology led
Content	Academic + PA	Academic + PA	Academic + PA	Academic + PA	Academic + PA	PA only
Cost	Free, $99 for GoNoodlePlus	Free	Free	K, 2nd, 3rd—$112 1st, 4th, 5th—$85 All grades—$591	Free	$99 for 11 DVDs
Activities Designed for Specific Grades	Yes	No	No	Yes	Yes	No
Grades	K–8th	No grades identified	No grades identified	K–5th	K–6th	No grades identified
Educational Content						
English-Language Arts	Yes	Yes	Yes	Yes	Yes	No
Health	Yes	Yes	No	Yes	Yes	No
Math	Yes	Yes	Yes	Yes	Yes	No
Science	Yes	Yes	No	Yes	Yes	No
Social Studies	Yes	Yes	Yes	Yes	Yes	No

Abbreviations: PA = Physical Activity, PAAC = Physical Activity Across the Curriculum, ABC = Activity Bursts in the Classroom.

**Table 2:** Demographics of Participating Schools and Teachers.

**School Demographics**	**School A**	**School B**	**School C**	**School D**	**Overall**
Number of students	492	385	591	706	2174
Number of teachers	40	31	38	55	164
Percent eligible for free or reduced lunch	100	100	46	100	86.5
Student–teacher ratio	12 to 1	12 to 1	16 to 1	13 to 1	13 to 1
Grades	PreK–5th	PreK–5th	K–4th	PreK–6th	n/a
**Teacher Demographics**					
Number of participating teachers	11	10	11	8	40
Years of teaching experience					
Less than 3	1	2	0	0	3
3–5	1	3	5	2	11
6–10	3	0	0	3	6
11–15	1	0	3	3	7
16–20	1	0	2	0	3
20–25	2	3	0	0	5
More than 25	2	2	1	0	5
Education background					
Bachelor’s degree	2	2	4	2	10
Master’s degree	8	7	7	5	27
Missing	1	1	0	1	3
Race					
African American	9	7	2	6	24
Non-Hispanic White	2	3	9	2	16

School demographics were attained from the National Commission for Education Statistics 2017–2018 academic year.

**Table 3:** MI Product Use.

	PAAC	GoNoodle	Take10	ABC for Fitness	Move for Thought	Instant Recess
Mean Uses/Week (Standard Deviation)	4.4 (1.4)	5.2 (1.6)	3.9 (1.5)	6.3 (2.0)	1.4 (1.7)	3.8 (1.1)
Median Uses/Week (Interquartile Range)	4.5 (3.0, 5.0)	4.3 (2.5, 6.9)	3.9 (2.5, 5.0)	4.7 (1.2, 7.1)	0.7 (0.0, 2.5)	3.8 (2.3, 5.0)
Mean Minutes of Planning Time/Use (Standard Deviation)	8.8 (5.1)	7.1 (3.3)	13.0(8.8)	13.7 (12.4)	15.8 (7.6)	8.8 (7.3)
Mean Duration of Use in Minutes (Standard Deviation)	11.0 (0.06)	6.0 (0.04)	12.0 (0.08)	11.0 (0.07)	15.0 (0.07)	8.0 (0.07)
Reason for Not Using a Product (% of teachers who stated reason for not using) ^a^						
Not enough time	100	100	100	62	54	71
Not enough space	-	-	-	8	-	-
Students don’t like it	-	-	-	8	15	21
Student misbehavior	-	-	-	15	15	7
Technical difficulties	-	-	-	-	46	21
Does not know how to use	-	-	-	23	31	

^a^ Percentages sum to greater than 100% because teachers cited multiple reasons for non-use on a given day; Abbreviations: PAAC= Physical Activity Across the Curriculum, ABC= Activity Bursts in the Classroom.

**Table 4:** Teacher Ratings by Theme.

	Subtheme	Scale	Number of Teachers	Number of Product Tests	Number of Schools	Overall	PAAC	GoNoodle	Take10	ABC	MFT	Instant Recess
Logistics	Time	+/−	27	32	4							
	Ease of use	+/−	30	77	4							
	Missing features	−	17	17	4							
	Alternative for outdoor activities	+	10	10	4							
Alignment w/Teaching Goals	Reinforces academics	+/−	30	36	4							
	Behavior management	+/−	18	21	3							
	Developmental Appropriateness	+/−	17	17	4							
	Refocus, breaks, transition	+	30	39	4							
	Customizable, flexible	+	12	12	3							
	Children’s health	+	10	11	3							
Student Needs and Interests	Student enjoyment	+/−	22	36	4							
	Different, new	+/−	18	23	3							
	Comfortable, class-specific interests	+	18	20	4							
	Movement break	+	27	27	4							

Green > 67%, Yellow 33%−66%, Red < 33% of teachers rated the product favorably. Gray = not mentioned. Scale: “+/−“ theme contained positive and negative comments, “+” theme contained only positive comments, “−” theme contained only negative comments. Number of teachers reflects the number of unique teachers who referenced the theme. Number of product tests reflects the number of products tests in which the theme was mentioned, max = 120. Number of schools reflects the number of schools in which the theme was mentioned. Abbreviations: PAAC = Physical Activity Across the Curriculum, MFT = Move for Thought.

## Appendix A. Post Product Survey

(1) Overall, I find [MI product] to be useful as a break from academic content.
(2) Overall, I find [MI product] to be useful for providing academic content.
(3) Overall, I find [MI product] to be useful for getting children physically active.
(4) I would continue to use [MI product] in my classroom.
(5) Overall, it was easy to learn how to use [MI product].
(6) Overall, [MI product] was easy to integrate into my classroom.
(7) Overall, I feel comfortable using [MI product] in my classroom.
(8) [MI product] was easy to use.
(9) It was easy to manage the classroom while using [MI product].
(10) Children were out of control while I was using [MI product].
(11) How satisfied are you with [MI product]?
(12) How likely are you to purchase [MI product]?
(13) What price would you be willing to pay to use [MI product] in your classroom?
(14) How likely are you to recommend [MI product] to other classroom teachers?
(15) If you no longer had access to [MI product] how likely would it be for you to recommend that your school purchase it again?

## Appendix B. Daily Diary Sheet

**Please Fill in the Information Below about Times when Your Class Was not in the Classroom Today:**				
Activity	Mark if went	Start Time	End Time	Comments
Recess	☐			
Lunch	☐			
PE	☐			
Related Arts	☐			
Other Out-of-Classroom Time	☐			

**If you DID USE ABC for Fitness Today, Please Answer the Questions Below**					

How long did you spend planning/preparing to use ABC for Fitness?					
	Hours			Minutes	

Of the features listed below which ones did you use during class with students today?					
Product/Features Used	Mark if used	Start Time	End Time	Academic content (ELA, Math, transition, break from academics, etc.)	Comments (please describe which activity you used)
Basic Activity Bursts	☐				
Enhanced Activity Bursts	☐				
Supplemental Information	☐				

**If you DID NOT USE ABC for Fitness Today, Please Answer this Question**					
Why did you not use the ABC for Fitness today?					
Children Misbehaving	☐	Children do not Like Product	☐	Not Enough Time	☐
Technical Difficulties	☐	Do not Know How to Use	☐	Not Enough Space	☐
Other *(Please Describe Other to the Right)*	☐				

## Appendix C. Focus Group Interview Questions

1. So, what did everyone like best about [[MI product]] after using it this past week?
  - Why did you like that feature of the product?
2. Was there anything that you didn’t like?
  - Why did you not like that feature of the product?
3. What did your students think of it?
  - What did they like? Not like? Why?
  - Were there any features that any of you had to modify when delivering content to your students? If yes, please explain which ones and why?
4. How easy was it to use the product?
  - What challenges did you encounter when integrating it into your curriculum?
  - Was there anything else that made it difficult to use?
5. Now that you have had a week of experience with it, how knowledgeable to you feel about [[MI product]]?
6. For those of you that did not use [[MI product]], could you please tell us why?
7. * Movement integration products have a variety of different features. For example, some include videos, equipment, or academic content. When considering the MI products you have used, what features would make a product more attractive for you to use?
8. Is there anything else anyone would like to share about your experiences, positive or negative?

* = This question was only asked during the final focus group (Product test #4) at each school.
